# Changes in the Dietary Habits of the Greek EPIC Cohort Participants during a 14-Year Follow-Up Period (1997–2011)

**DOI:** 10.3390/nu12072148

**Published:** 2020-07-19

**Authors:** Nikolaos Skourlis, Ioannis Patsis, Georgia Martimianaki, Eleni Peppa, Antonia Trichopoulou, Klea Katsouyanni

**Affiliations:** 1 Hellenic Health Foundation, 11527 Athens, Greece; ptsyannis@gmail.com (I.P.); g.martimianaki@hhf-greece.gr (G.M.); e.peppa@hhf-greece.gr (E.P.); atrichopoulou@hhf-greece.gr (A.T.); 2 National and Kapodistrian University of Athens, 11527 Athens, Greece; kkatsouy@med.uoa.gr

**Keywords:** food questionnaire, nutritional behavior, follow-up dietary data, EPIC, ordinal regression

## Abstract

The aim of this study is to evaluate the changes in the nutritional behavior of the Greek EPIC (European Prospective Investigation into Cancer and Nutrition) cohort participants regarding the consumption of basic food groups, during a 14-year period (1997–2011). In the Greek segment of the EPIC cohort study (EPIC-Greece), the changes in dietary habits of 23,505 participants regarding several food items/groups (vegetables, legumes, fruits, nuts, dairy, cereal, meat, fish/seafood, olive oil) were recorded repeatedly over time and compared to the baseline assessment (1994–1997), using a short, qualitative, follow-up questionnaire. Descriptive statistics were used to study the trends in nutritional behavior over time and ordinal logistic regression models to study the associations between the ordered responses of the questionnaire and sociodemographic and health factors. More participants reported an increase rather than a decrease in the consumption of vegetables, fruits, fish/seafood, whilst the inverse was observed for dairy products, nuts, cereals, and meat. No prevailing trend was noted for legumes and olive oil. Factors such as being female and having high education relate to more positive (healthy) changes in nutritional behavior. There seems to be primarily a change to a more healthy nutritional behavior of the EPIC-Greece participants over the follow-up period, with different participant subgroups presenting different degrees of nutritional changes.

## 1. Introduction

Dietary habits vary over time [1,2,3,4] and are correlated with a wide range of individual characteristics, such as biological, demographic, psychological, situational and socio-cultural on an interpersonal level [5]. The association between dietary behavior and health has been studied intensively over the last decades, the literature in the area of nutritional epidemiology is very extensive and the evidence of association can vary from weak to strong depending on the health outcome studied [6,7,8]. Therefore, it is of high public health importance to recognize trends in the dietary habits of a population. Due to the heterogeneity of dietary habits between different population subgroups, it is also of high priority to study the nutritional trends in association with socioeconomic, demographic and health factors as part of well-designed policy plans and strategies to address nutritional trends towards more unhealthy patterns of consumption, and specify the subgroups that should be the main focus of these policies.

The National Diet and Nutrition surveys/Food Observation surveys [1,9,10] and long-term cohort studies [11,12,13] aim to assess nutritional trends either in representative samples of the national population or among cohort participants. Among the cohort studies with repeated assessment of dietary habits, most use a unique quantitative questionnaire (i.e., the same as baseline) or a shorter version of the baseline questionnaire for follow-up information. It is less common to use qualitative follow-up questions to evaluate changes in the dietary habits of the study population [14]. Using a qualitative dietary follow-up questionnaire has the advantage of being simpler to administer on a large scale. With the use of appropriate statistical models, the information provided by qualitative follow-up questionnaires can be used to study the association between a large number of sociodemographic, somatometric and health status factors, as well as for the assessment of nutritional trends.

This less common qualitative questionnaire approach was explored in order to meaningfully capture the long-term changes in the dietary preferences of the EPIC-Greece cohort participants, who were asked about dietary changes compared to baseline during follow-up. The aim of this paper is to present those trends in dietary habits in a qualitative manner and study their association with basic demographic, socioeconomic, somatometric factors and pre-existing health conditions at baseline, such as heart attack, diabetes (DM), stroke, cancer, peptic ulcer, high blood pressure and hypercholesterolemia, regarding the consumption of basic food items/groups, namely, vegetables, legumes, fruits, nuts, dairy, cereal, meat, fish/seafood, and olive oil, through ordinal logistic regression models.

## 2. Materials and Methods

### 2.1. The EPIC-Greece Study

European Prospective Investigation into Cancer and Nutrition (EPIC) is a multicenter prospective cohort study in 10 European countries, investigating the association of several risk factors, with a special focus on diet with cancer and other health outcomes [15]. Many publications of this major study have contributed to our knowledge of nutrition and health [8,16,17]. The Greek segment of EPIC started in 1994, after the successful implementation of a pilot study during the period 1991–1993. The recruitment of volunteers was completed in 1999. All volunteers gave their informed consent for inclusion before they participated in the study. The study was conducted in accordance with the Declaration of Helsinki. The EPIC-Greece cohort consists of 28,572 adults, aged between 25 up to 86 years old, recruited from all over Greece on a voluntary basis, and representing a broad range of sociodemographic factors. To enhance the response rate, a campaign informing potential participants was conducted via television, radio and announcements at the local community centers. At enrollment, detailed information on the participants’ demographic and socioeconomic characteristics, physical activity anthropometric measurements, medical and reproductive history was recorded via a face-to-face interview with the volunteer at the local health centers. Dietary intake at baseline was interviewer-administered via a detailed semi-quantitative food frequency questionnaire (FFQ) [18].

### 2.2. Follow-Up

After the initial enrollment, between 1994 and 1997, 26,579 participants were followed-up every 3–4 years in order to update the information on lifestyle factors, including diet, and on the health status. There were no exclusion criteria. The follow-up of participants between 1997 and 2011 was conducted mainly by telephone interviewer-administered questionnaires (92.34% of the interviews) and to a small extend by mail (7.15%) or by personal interviews (0.51%). When the participant was not found, information on vital status was obtained by the next of kin. Information on the occurrence of cancer and cardiovascular diseases was confirmed by hospital records.

### 2.3. The Follow-Up Dietary Questionnaire (FU-DQ)

In the first follow-up period (1997–2002), the questionnaire did not include questions on food consumption. From the second follow-up onwards (after 2002), a dietary follow-up questionnaire (FU-DQ) was designed and administered to 23,505 participants. The FU-DQ included questions on the consumption of the following food items/groups: potatoes, vegetables, legumes, fruits, dairy, low-fat dairy, nuts, cereal, total red meat, lamb/goat, poultry, fish/seafood, eggs, olive oil, seeds oil, margarine, butter, sugar and confectionery products, and alcoholic and non-alcoholic beverages. For all food items/groups excluding alcoholic beverages, individuals were asked to qualitatively report the change in consumption when compared to their consumption at baseline. The questions regarding the change in food consumption included seven pre-specified, categories: “No consumption (None)”, “Much less”, “Less”, “The same”, “More”, “Much”, “More” and “Do not remember”.

### 2.4. Study Sample

The EPIC follow-up is a dynamic, continuous process that started in 1997 while the follow-up dietary questionnaire (FU-DQ) started in 2002. In the present analysis we include the dietary follow-up until 2011. Within that period of 14 years, each participant was interviewed approximately every 3–4 years. The first follow-up period was in 1997–2002, the second in 2002–2007 (first FU-DQ period) and the third was in 2007–2011 (second FU-DQ period).

### 2.5. Statistical Analysis

Stata software version 11 was used [19] for the derivation of frequency tables, summary statistics, figures, as well as for the logistic regression models of the analysis. The changes in consumption were expressed as frequencies, classifying the potential answers as “Less/Much less”, “The same”, and “More/Much More”, for the consumption of vegetables, legumes, fruits, nuts, dairy products, cereal, meat, fish/seafood and olive oil in comparison to their baseline consumption assessment. Ordinal logistic regression models were used to assess the association of several factors as measured at baseline (quartiles of FFQ consumption, age, gender, education, body mass index (BMI), heart attack, diabetes, stroke, peptic ulcer, primary cancer, hypercholesterolemia and high blood pressure), with the odds ratio (OR) of a participant of a specific covariate pattern reporting higher or lower consumption of a food item/group at baseline compared to a participant of another covariate pattern (e.g., males versus females). For each food item/group, the FU-DQ responses were classified into a naturally ordered response variable with three levels (first level: “Much Less/Less”, second level: “Same”, third level: “More/Much More”) and separate analyses were conducted for the first and second FU-DQ periods.

An initial significance level of *a* = 0.05 was used for all statistical analyses. However, a second significance level, derived from the Bonferroni correction, is also provided in order to take into account the multiple statistical hypothesis testing. The value of the Bonferroni-corrected significance level is a/m=a/288=0.00017, where *m* is the total number of the tested hypotheses. Only results statistically significant on the Bonferroni-corrected significance level will be addressed (noted with **).

It should be noted that in the ordinal logistic models, for a one unit increase in one factor (e.g., female (gender = 1) versus male (gender = 0)), the odds of reporting “Much More/More” versus reporting “Same” and “Less/Much Less” categories are OR times greater (or lower), given that the other variables are held constant in the model. Likewise, the same interpretation holds for the odds of reporting “Much More/More” and “Same” versus reporting “Less/Much Less”. Due to the great volume of results, the interpretations of the models will be shorter than the above example.

Participants that had no interview during a FU-DQ period, were not taken into account for the particular period. In addition, if a participant happened to have more than one follow-up interview during the same period, only his/her first interview for the specific FU-DQ period was taken into consideration. The statistical analysis approach is a complete case analysis, meaning that all participants with missing values on a food item/group during an FU-DQ period, are excluded from the statistical analysis during that specific period. Sources of loss to follow up are deemed discontinued participation due to the inability to further engage with the participant as a result of change in contact details or death.

## 3. Results

Overall, 28,572 individuals (11,953 men and 16,619 women) were recruited in the EPIC-Greece study, from which 26,579 (10,943 men and 15,645 women) took part in the follow-up process and 23,505 took part in the FU-DQ. In total, 6,009 participants completed only one FU-DQ questionnaire (25.6%), 15,225 participants completed two FQ-DQ questionnaires (64.7%) and 2,271 participants completed all three questionnaires (9.7%). The number of individuals of EPIC-Greece cohort study that participated in the 1^st^ and 2^nd^ FU-DQ period is presented in Table 1. Table 2; Table 3 provide the descriptive statistics for selected baseline characteristics of the EPIC-Greece participants and the quartiles of baseline FFQ consumption of the food groups/items of interest.

### 3.1. Qualitative Assessment of Trends in Nutritional Behavior

**Figure 1:**
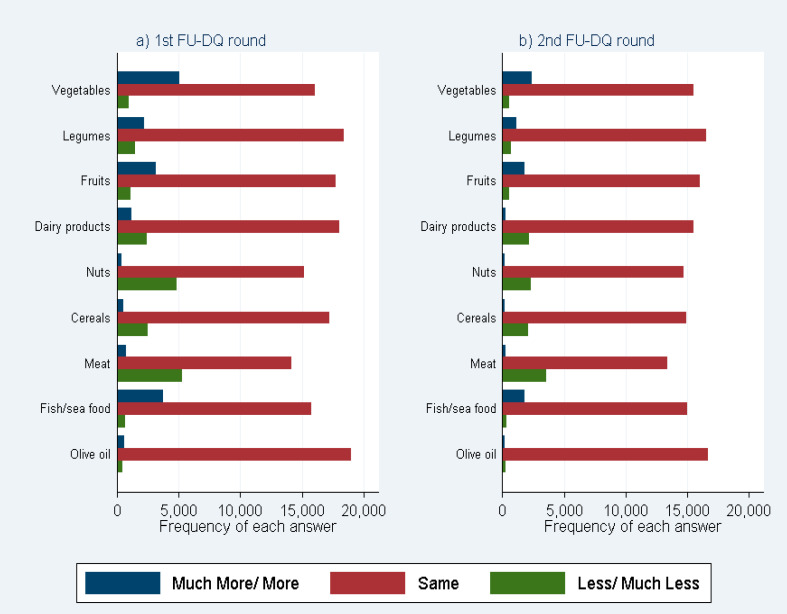
Bar plots for frequency of answers over the food items/groups of interest for the two rounds of the FU-DQ questionnaires: (**a**) first FU-DQ period; (**b**) second FU-DQ period.

Figure 1 depicts the frequencies of the answers regarding food item/group for the first FU-DQ period (Figure 1a) and the second FU-DQ period (Figure 1b). Based on these figures, we qualitatively assess the crude trends in the nutritional behavior of the EPIC-Greece participants. Figures S1 and S2 in the complementary material show the frequencies of answers regarding food item/group over quartiles of baseline FFQ consumption within each food item/group for the first (Figure S1) and second FU-DQ period (Figure S2). These figures provide further insight of whether baseline consumption (baseline FFQ quartile) relates to different frequency of each answer. In addition, a detailed frequency table of each FU-DQ answer category regarding consumption of the food items/groups of interest comparative to intake at baseline by FU-DQ period, is available in the complementary material.

In Figure 1, it is explicitly shown that the answer with the highest frequency in all cases is the answer “The same”. However, based on the frequencies of the answers “More”–“Much More” and “Less”–“Much Less”, we can categorize the food items/groups into three categories: (a) food items/groups where more participants reported an increase rather than a decrease in consumption (vegetables, fruits and fish/seafood); (b) food items/groups where the inverse happened, that is, where more participants reported a decrease rather than an increase in consumption (dairy products, nuts, cereals, meat products); and (c) food items/groups where the frequencies of participants reporting an increase in consumption were roughly equal to those reporting a decrease (legumes, olive oil). It should be noted that, generally, opposite to dairy products, low-fat dairy products belong to the first category, where more participants reported an increase rather than a decrease in consumption (not depicted in the figure).

### 3.2. Association of Dietary Changes with Socioeconomic, Demographic and Health Factors

Table 4 and Table 5 provide the odds ratios of all the ordinal logistic models that were fit for each food item/group separately over the first FU-DQ (Table 4) and second FU-DQ period (Table 5).

Table 4 shows the model results regarding the first FU-DQ period (2002–2007). Belonging to a higher quartile of baseline FFQ consumption is correlated with a relevant increase in the odds of reporting higher consumption of all food items/groups, except olive oil and nuts (with the exception the participants of the top FFQ baseline quartile). Sensitivity analyses show the presence of a trend (*p* < 0.0001) for all foods except olive oil, meaning that, in general, the higher the quartile of initial FFQ consumption, the greater the chance of reporting increased consumption. A 5-year increase in baseline age is correlated with decreased odds of higher consumption of vegetables, legumes and fruits. Being female is correlated with lower odds of reporting higher consumption of meat and nuts than men, but also higher odds for vegetables, fish/sea food and dairy products. Education level seems to be strongly associated with the responses for most food items/groups. Having a medium or high education level is correlated with increased odds of reporting higher consumption of vegetables, fruits and fish/sea food when compared to having a low educational level, and decreased odds of reporting higher consumption of meat. Having a high educational level in particular is also correlated with increased odds of reporting higher consumption of legumes and olive oil. Being overweight (25 < BMI ≤ 30) or obese (BMI ≥ 30) is correlated with decreased odds of reporting higher consumption of nuts, cereals and olive oil.

Having a heart attack history at baseline is correlated with a decrease in odds of reporting higher consumption of meat and dairy products and increased odds of reporting higher consumption of fruits and fish/sea food compared to healthy individuals. However, those results are not statistically significant on the Bonferroni corrected significance level. Having diabetes mellitus at baseline is associated with increased odds of higher vegetable consumption and decreased odds of reporting higher consumption of fruits, legumes, cereals and nuts, showing that the diabetic participants altered their behavior towards these food groups most likely as a result of the diet restrictions imposed by the condition. History of cancer, peptic ulcer and high blood pressure at baseline do not show any strong associations with the responses of the first FU-DQ period on the Bonferroni corrected level of 0.00017. Hypercholesterolemia appears to be correlated with increased odds of reporting higher vegetable, fruit and fish/sea food consumption and decreased odds of reporting higher meat, nut and dairy product consumption.

Table 5 shows the model results regarding the second FU-DQ period (2007–2011). Belonging to higher quartiles of baseline FFQ consumption is correlated with increased odds of reporting higher consumption of vegetables, meat, fish /sea food and decreased odds of reporting olive oil consumption when compared to participants of the lower FFQ quartile at baseline. Sensitivity analyses show the presence of a trend (p < 0.0004) of consumption of vegetables, fruits, meat, fish/seafood and olive oil, meaning that for half of the food items/groups, the higher the quartile of initial FFQ consumption, the greater the chance of reporting increased consumption. A 5-year increase in age at baseline is correlated with decreased odds of higher consumption of vegetables, legumes, fruits and fish/sea food and increased odds of dairy product consumption. Being female is correlated with lower odds than males of reporting higher consumption of legumes and dairy products when compared to baseline. Having a medium or high education level is correlated with increased odds of reporting higher consumption of fruits and fish/sea food compared to having a low educational level, and decreased odds of reporting higher consumption of meat, which is similar to the first FU-DQ. In particular, having a high educational level is also correlated with increased odds of reporting higher consumption of legumes and vegetables and decreased odds of reporting consumption of dairy products. Being overweight or obese correlates with decreased odds of reporting higher consumption of cereals than baseline when compared to being of normal weight. In specific, being obese is also associated with decreased odds of reporting higher consumption of nuts and dairy products than baseline. Among the health condition variables, only diabetes and hypercholesterolemia showed statistically significant correlations with changes in nutritional behavior at the Bonferroni corrected significance level. In particular, having diabetes at baseline is correlated with decreased odds of reporting higher consumption than baseline for fruits, legumes and cereals, which is in accordance with the results of the first FU-DQ period analysis. Last but not least, as in the first FU-DQ period, hypercholesterolemia appears to be correlated with a decreased chance of reporting higher meat consumption.

## 4. Discussion

This study aimed to assess the changes in the nutritional behavior of the Greek EPIC cohort participants over time and study the association of these changes with demographic, socioeconomic and health factors measured at baseline.

Among the participants of EPIC-Greece reporting a change in the consumption of a food item/group, more participants reported an increase in the consumption of vegetables, fruits, fish/seafood and low-fat dairy products and a decrease in dairy products (general), meat and meat products, nuts and cereals, while those reporting increased consumption of olive oil and legumes were roughly equal to those reporting a decrease. These results are indicative of an overall tendency towards a healthier diet. Among the characteristics of the profile of participants implementing changes towards a healthier nutritional profile, higher education level was associated with significant changes, as well as female gender.

Among the health conditions considered, participants with diabetes and high blood cholesterol at baseline were reporting changes expected for their conditions (such as increased vegetable and decreased fruit consumption for diabetic subjects, and increased vegetable, fruits and fish/sea food and decreased meat, nut and dairy product consumption for those with high blood cholesterol). A consistent finding was that those reporting higher consumption at baseline reported also more pronounced changes.

These results from EPIC-Greece can be compared with those of other cohort studies in Europe [11,12,13]. In the SUN study [12] (Spain, 1999–2013), the results show an increase in the consumption of vegetables, fruits, cereals and nuts; stable consumption of fish and olive oil; a mixed trend for dairy products (increase in low-fat products, decrease in high-fat products); and a decrease in the consumption of legumes and meat/meat products. This is largely consistent with our findings, which also report increasing trends for vegetable and fruit consumption, an unchanged consumption of olive oil and a decrease in meat consumption. However, while EPIC-Greece reports a decrease in cereals, nuts and dairy products and a stable consumption of legumes, SUN reports an increase in the consumption of nuts and cereals, mixed trends in dairy product consumption and a decrease in legume consumption. It should be noted that the SUN study cohort population consists of younger participants with a mean age of 35 years at baseline, while the mean age of EPIC-Greece participants was 57 years at recruitment. In addition, all participants in the SUN cohort are of high educational level (university graduate students and health care professionals), while only 17.2% of the EPIC-Greece participants have a post-secondary education degree. Our finding that higher educational level influences dietary habits may be the reason behind some rather minor differences between our study and SUN.

A similar study whose results can be compared to ours has been reported by the Danish MONICA project, even though the follow-up period did not cover the same calendar period as our study [11,12,13]. The cohort population of this study was drawn from random, age-stratified samples of 30, 40, 50 and 60 years of age at recruitment and had a 10 year follow-up period (1982–1992). Reported changes in dietary habits from the MONICA project are consistent with the results of EPIC-Greece, reporting an increase in vegetables, fruits, fish (in men) and a decrease in meat and dairy products. However, the MONICA study reports different trends for subtypes of cereals, and refers to “plant-based oils” without explicitly mentioning olive oil, and there is no information regarding nuts.

Although the above studies are not identical as far as mean age at baseline or their calendar period, it is important to note the fact that in all studies, the participants report an overall tendency of healthier nutritional habits with the passage of time [11,12,13].

For the association between the demographic factors and baseline consumption (FFQ quartiles) with the changes in consumption during follow-up, Andrade L. et al. [14], using data from the SUN study, provide broadly comparable results using a composite score of “healthy eating attitudes” based on the responses to 10 qualitative questions. In this study, female gender and older age at baseline are positively associated with an increase in score, while BMI greater that 25 at baseline does not seem to have an association with the score. Our findings are consistent in indicating that female gender is associated with a change towards a healthier consumption pattern, and that baseline BMI is not consistently associated with changes in nutritional behavior. However, age in our study is associated with smaller consumption of all food groups.

A higher quartile of Mediterranean diet score at baseline was associated with a greater positive change in nutritional attitude in the SUN participants after 10 years of follow-up compared to those belonging to the first quartile. In our study, we used FFQ baseline quartiles of consumption in the analysis, showing that the higher the quartile of baseline consumption, the higher the odds of reporting higher consumption than baseline for almost all food items/groups. The results from the two studies, while not directly comparable, do indicate that higher quartiles of consumption and Mediterranean diet score at baseline relate with reporting higher changes in consumption and nutritional behavior.

In our study, comparing the results from the two FU-DQ periods showed that during the second FU-DQ period, the general pattern of changes and their determinants remain consistent, but the reported changes are less pronounced. As out participants were always asked to compare with the baseline period, this can be a memory effect as time becomes more distant.

This study has a number of strengths and several limitations. EPIC-Greece is a large cohort study, including more than 28,500 participants. The large majority of the initial participants completed at least one follow-up dietary questionnaire during the period of 2002 to 2011. The participants were recruited from all over the country, thus covering a broad range of socio-demographic factors [8,20]. However, the information on dietary changes is qualitative and was obtained through a short follow-up questionnaire, so the true extent of the reported changes was not possible to be quantitatively assessed. In such case, there can be misreporting because of recall bias or other perception fallacies. Another issue is that not all participants participated in each one of the FU-DQ periods. Last but not least, albeit a large sample, the EPIC-Greece cohort was not designed to be a representative sample of the Greek population. Therefore, it would be preferable to avoid generalizing the trends observed in this study to the overall population. However that results on the demographic, socioeconomic and health profile of people following different dietary patterns over time provides valid and useful results for public health policies.

## 5. Conclusions

For the participants of the EPIC-Greece study, the reported dietary changes during follow-up in comparison to baseline suggest, for most of the food groups, a trend towards a healthier diet. However, the change in the nutritional behavior is not the same among the different subgroups, indicating that targeted health policy plans and strategies on specific sub- population groups should be encouraged. With dietary habits and their trends significantly affecting health outcomes both on a population and an individual level, such recordings and analysis of these trends are useful in further investigating the relationship between health and nutrition.

## Figures and Tables

**Table 1:** Participation in each follow-up dietary questionnaire FU-DQ period.

EPIC FU-DQ Period	Date Range of Each FU-DQ Period	Number of Participants in Each FU-DQ Period	Mean Age of Participants in Each FU-DQ Period
1st FU-DQ	15/11/2002–24/06/2007	22,220	61 ± 12
2nd FU-DQ	25/06/2007–02/05/2011	18,452	65 ± 11.5

**Table 2:** Baseline characteristics of the participants of the EPIC-Greece study for the explanatory variables of interest.

Demographic Characteristics		
Age at baseline (years), Mean ± SD *	52.96 (±12.68)	
Gender	Males (N, %)	11,954 (41.8%)
	Females (N, %)	16,618 (58.2%)
Education level **	Low level (N, %)	5884 (21%)
	Medium level (N, %)	17,330 (61.8%)
	High level (N, %)	4816 (17.2%)
BMI ** (kg/m^2^)	<25 (N, %)	6293 (21.9%)
	25–30 (N, %)	12,139 (42.5%)
	>30 (N, %)	10,170 (35.6%)
Health status variables at baseline **		
Heart attack history (N, %)	538 (1.9%)	
Diabetes history (N, %)	1989 (7%)	
Cancer history (N, %)	486 (1.7%)	
Stroke history (N, %)	327 (1.14%)	
Peptic Ulcer history (N, %)	1281 (4.48%)	
High Blood Cholesterol (N, %)	6852 (23.98%)	

* SD: standard deviation. ** 542 participants (1.9%) with missing values for education level, BMI (body mass index) and the health status variables.

**Table 3:** Baseline food group consumption of the participants of the EPIC-Greece study for the food items/groups of interest.

	Food Frequency Questionnaire (FFQ) Quartiles of Consumption of the Relevant Food Items at Baseline (g/day)		
Food item/group	1st quartile	Median	3rd quartile
Vegetables	4.01	514.8	650.1
Legumes	4.26	7.05	12.15
Fruits	232.9	334	450.3
Dairy products	111.8	193.2	295.8
Nuts	1.12	5.36	8.25
Cereals	115	150.7	289.9
Meat	42	64.7	94
Fish/seafood	12.9	20.6	30
Olive oil	33.06	46.5	60.2

**Table 4:** Ordinal logistic regression performed on the food items/groups of interest for the 1^st^ FU-DQ. The ordinal response variable has three categories (“Much More/More”, “Same”, or “Less/Much Less”). The estimated measure is the Odds Ratio (OR). Confidence intervals of the estimations are also given (CI).

1st FU-DQ Period	Vegetables	Legumes	Fruits	Dairy Products	Nuts	Cereals	Meat	Fish/Sea Food	Olive Oil
	OR (95% CI)	OR (95% CI)	OR (95% CI)	OR (95% CI)	OR (95% CI)	OR (95% CI)	OR (95%CI)	OR (95% CI)	OR (95% CI)
FFQ 1st q. (ref. cat)	1 (-)	1 (-)	1 (-)	1 (-)	1 (-)	1 (-)	1 (-)	1 (-)	1 (-)
FFQ 2nd quartile	1.27 * (1.17–1.39)	1.25 ** (1.13–1.38)	1.26 ** (1.14–1.39)	1.25 ** (1.13–1.38)	1.07 (0.98–1.17)	1.33 ** (1.2–1.46)	1.21 ** (1.11–1.31)	1.23 ** (1.12–1.35)	0.89 (0.76–1.05)
FFQ 3rd quartile	1.43 ** (1.31–1.56)	1.46 ** (1.32–1.62)	1.46 ** (1.32–1.61)	1.5 ** (1.35–1.65)	1.09 (1–1.19)	1.5 ** (1.35–1.66)	1.41 ** (1.29–1.53)	1.43 ** (1.31–1.57)	0.95 (0.81–1.12)
FFQ 4th quartile	1.76 ** (1.61–1.92)	1.56 ** (1.41–1.73)	1.69 ** (1.53–1.86)	1.55 ** (1.4–1.72)	1.34 ** (1.22–1.48)	1.66 ** (1.49–1.85)	1.59 ** (1.46–1.74)	1.9 ** (1.73–2.09)	0.87 (0.73–1.03)
Baseline age	0.93 ** (0.92–0.95)	0.92 ** (0.91–0.94)	0.95 ** (0.93–0.96)	1 (0.98–1.01)	1.01 (0.99–1.03)	0.97 * (0.95–0.99)	0.96 ** (0.94–0.97)	0.98 * (0.96–0.99)	0.96 * (0.93–0.99)
Gender									
Males (ref. cat)	1 (-)	1 (-)	1 (-)	1 (-)	1 (-)	1 (-)	1 (-)	1 (-)	1 (-)
Females	1.21 ** (1.13–1.29)	0.88 * (0.82–0.95)	1.04 (0.97–1.12)	1.25 ** (1.16–1.35)	0.86 ** (0.81–0.92)	0.95 (0.87–1.03)	0.85 ** (0.8–0.91)	1.21 ** (1.13-1.3)	0.87 * (0.77-0.98)
Education									
Low education	1 (-)	1 (-)	1 (-)	1 (-)	1 (-)	1 (-)	1 (-)	1 (-)	1 (-)
Medium education	1.33 ** (1.22–1.46)	1.11 * (1.01–1.23)	1.26 ** (1.14–1.39)	1.04 (0.94–1.15)	0.87 * (0.8–0.96)	0.92 (0.83–1.02)	0.77 ** (0.71–0.83)	1.5 ** (1.36–1.65)	0.98 (0.83–1.15)
High education	2.48 ** (2.21–2.78)	1.38 ** (1.2–1.58)	2.07 ** (1.82–2.36)	1.1 (0.96–1.27)	0.81 * (0.72–0.92)	0.88 (0.77–1.02)	0.4 ** (0.36–0.45)	2.73 ** (2.41–3.08)	1.54 ** (1.24–1.92)
BMI group									
<25 (ref.cat)	1 (-)	1 (-)	1 (-)	1 (-)	1 (-)	1 (-)	1 (-)	1 (-)	1 (-)
25–30	1.05 (0.97–1.14)	1.11 * (1.01–1.22)	1.1 * (1.01–1.2)	0.89 * (0.81–0.99)	0.76 ** (0.7–0.83)	0.76 ** (0.69–0.85)	0.93 (0.86–1.01)	1.03 (0.95–1.13)	0.81 * (0.69–0.94)
>30	1.04 (0.96–1.14)	1.12 * (1.01–1.24)	1.08 (0.98–1.19)	0.83 * (0.75–0.92)	0.72 ** (0.65–0.78)	0.61 ** (0.55–0.68)	0.96 (0.88–1.04)	1.01 (0.92–1.1)	0.69 ** (0.59–0.81)
Heart attack history	1.27 (0.99–1.62)	1.23 (0.92–1.64)	1.42 * (1.08–1.85)	0.72 * (0.56–0.94)	0.98 (0.76–1.26)	0.86 (0.66–1.13)	0.78 * (0.62–0.97)	1.5 * (1.17–1.92)	1.25 (0.79–1.98)
Diabetes history	1.33 ** (1.17–1.51)	0.82 * (0.7–0.95)	0.63 ** (0.54–0.74)	0.94 (0.81–1.09)	0.77 ** (0.68–0.87)	0.35 ** (0.31–0.4)	1.07 (0.95–1.21)	1.02 (0.89–1.17)	0.71 * (0.56–0.89)
Cancer history	1.07 (0.85–1.35)	0.98 (0.74–1.29)	1.09 (0.84–1.41)	1.11 (0.84–1.47)	1.16 (0.9–1.48)	1.09 (0.83–1.45)	0.81 (0.66–1.01)	1.13 (0.89–1.43)	1.34 (0.87–2.05)
Stroke history	1.15 (0.84–1.58)	0.79 (0.55–1.13)	1 (0.71–1.43)	0.83 (0.59–1.18)	0.81 (0.59–1.11)	0.61 * (0.45–0.85)	0.84 (0.63–1.12)	1.01 (0.72–1.41)	0.68 (0.39–1.19)
Peptic Ulcer history	0.81 * (0.7–0.95)	0.75 * (0.63–0.9)	1.02 (0.86–1.2)	0.88 (0.74–1.05)	0.9 (0.77–1.05)	0.87 (0.73–1.03)	1.01 (0.87–1.16)	1.17 (1–1.36)	0.86 (0.65–1.13)
High Blood Pressure	0.99 (0.91–1.07)	0.97 (0.88–1.06)	1 (0.92–1.1)	0.88 * (0.81–0.97)	0.89 * (0.82–0.96)	0.91 * (0.83–0.99)	0.99 (0.92–1.07)	0.95 (0.88–1.04)	0.94 (0.81–1.09)
High Blood Cholesterol	1.23 ** (1.15–1.33)	1.15 * (1.06–1.26)	1.2 ** (1.11–1.3)	0.76 ** (0.7–0.83)	0.79 ** (0.74–0.85)	0.9 * (0.83–0.98)	0.81 ** (0.76–0.87)	1.18 ** (1.1–1.28)	0.96 (0.84–1.1)

* Statistical significance at *a* = 0.05 significance level, ** statistical significance at *a’* = 0.00017 level after Bonferroni correction.

**Table 5:** Ordinal logistic regression performed on the food items/groups of interest for the 2^nd^ FU-DQ. The ordinal response variable has three categories (“Much More/More”, “Same”, or “Less/Much Less”). The estimated measure is the Odds Ratio (OR). Confidence intervals of the estimations are also given (CI).

2nd FU-DQ Period	Vegetables	Legumes	Fruits	Dairy Products	Nuts	Cereals	Meat	Fish/Seafood	Olive Oil
	OR (95% CI)	OR (95% CI)	OR (95% CI)	OR (95% CI)	OR (95% CI)	OR (95% CI)	OR (95% CI)	OR (95% CI)	OR (95% CI)
FFQ 1st q. (ref. cat)	1 (-)	1 (-)	1 (-)	1 (-)	1 (-)	1 (-)	1 (-)	1 (-)	1 (-)
FFQ 2nd quartile	1.13 * (1–1.26)	1.13 (0.98–1.29)	1.03 (0.91–1.17)	1.04 (0.92–1.18)	1.05 (0.93–1.18)	1.02 (0.91–1.15)	1.03 (0.94–1.14)	1.11 (0.97–1.25)	0.57 ** (0.44–0.73)
FFQ 3rd quartile	1.26 ** (1.12–1.42)	1.16 * (1.01–1.33)	1.05 (0.93–1.19)	1 (0.89–1.13)	1 (0.89–1.13)	1.03 (0.91–1.16)	1.24 ** (1.12–1.37)	1.18 * (1.04–1.34)	0.48 ** (0.37–0.62)
FFQ 4th quartile	1.35 ** (1.21–1.52)	1.24 * (1.08–1.42)	1.25 * (1.1–1.41)	1.03 (0.91–1.16)	1.09 (0.96–1.24)	1.12 (0.99–1.28)	1.3 ** (1.17–1.45)	1.39 ** (1.23–1.57)	0.45 ** (0.3 0.58)
Baseline age	0.89 ** (0.87–0.91)	0.89 ** (0.87–0.91)	0.91 ** (0.89–0.93)	1.05 ** (1.02–1.07)	1.03 * (1.01–1.05)	1.03 * (1.01–1.05)	0.99 (0.97–1.01)	0.94 ** (0.92–0.96)	0.99 (0.94–1.04
Gender									
Males (ref.cat)	1 (-)	1 (-)	1 (-)	1 (-)	1 (-)	1 (-)	1 (-)	1 (-)	1 (-)
Females	0.97 (0.89–1.05)	0.8 ** (0.72–0.88)	0.94 (0.86–1.03)	0.8 ** (0.72–0.87)	1.01 (0.93–1.11)	0.95 (0.86–1.04)	0.97 (0.9–1.05)	0.97 (0.89–1.07)	0.89 (0.74–1.07)
Education									
Low education (ref. cat)	1 (-)	1 (-)	1 (-)	1 (-)	1 (-)	1 (-)	1 (-)	1 (-)	1 (-)
Medium education	1.25 * (1.11–1.41)	1.22 * (1.06–1.4)	1.37 ** (1.2–1.56)	0.84 * (0.74–0.95)	1 (0.89–1.12)	0.99 (0.88–1.12)	0.83 ** (0.75–0.91)	1.5** (1.31–1.71)	1.05 (0.83–1.34)
High education	1.86 ** (1.59–2.17)	1.91 ** (1.58–2.29)	2.02 **(1.7–2.39)	0.72** (0.61–0.85)	1.12 (0.95–1.32)	0.98 (0.83–1.15)	0.63 ** (0.55–0.72)	2.18** (1.84–2.59)	0.92 (0.66–1.28)
BMI group									
<25 (ref. cat)	1 (-)	1 (-)	1 (-)	1 (-)	1 (-)	1 (-)	1 (-)	1 (-)	1 (-)
25–30	1.09 (0.97–1.21)	1.03 (0.91–1.17)	1.17 * (1.04–1.32)	0.8 * (0.71–0.91)	0.85 * (0.76–0.96)	0.69 ** (0.61–0.79)	0.93 (0.84–1.02)	1.12 (0.99–1.26)	0.97 (0.76–1.22)
>30	1.14 * (1.01–1.27)	0.98 (0.85–1.13)	1.17 * (1.03–1.33)	0.78 ** (0.69–0.89)	0.7 ** (0.62–0.8)	0.47 ** (0.41–0.54)	0.87 * (0.79–0.96)	1.19 * (1.05–1.35)	0.8 (0.62–1.03)
Heart attack history	0.72 (0.5–1.05)	1.08 (0.71–1.64)	0.86 (0.58–1.27)	0.94 (0.65–1.37)	1.34 (0.91–1.98)	1.35 (0.91–1.99)	1.04 (0.78–1.39)	1.11 (0.76–1.6)	1.1 (0.53–2.32)
Diabetes history	0.98 (0.82–1.18)	0.6 ** (0.49–0.74)	0.63 ** (0.51–0.77	0.86 (0.72–1.03)	0.74 * (0.63–0.88)	0.41 ** (0.35–0.47)	0.85 * (0.74–0.98)	0.88 (0.72–1.07)	0.75 (0.53–1.08)
Cancer history	0.74 (0.52–1.05)	0.87 (0.58–1.3)	0.86 (0.59–1.24)	0.99 (0.7–1.4)	1.28(0.89–1.85)	1.37 (0.94–2.01)	1.21 (0.9–1.61)	1.11 (0.78–1.57)	0.87 (0.44–1.72)
Stroke history	0.55 * (0.35–0.88)	0.51 * (0.32–0.82)	0.62 (0.38–1.01)	0.94 (0.59–1.5)	1.02 (0.65–1.58)	1.22 (0.77–1.94)	1.13 (0.77–1.64)	0.81 (0.49–1.34)	0.34 * (0.17–0.7)
Peptic Ulcer history	1.08 (0.88–1.32)	1.02 (0.81–1.3)	1 (0.81–1.25)	1.06 (0.85–1.32)	0.88 (0.72–1.07)	0.89 (0.73–1.09)	0.87 (0.74–1.03)	1.13 (0.92–1.4)	0.71 (0.47–1.06)
High Blood Pressure	0.95 (0.85–1.05)	0.83 * (0.73–0.94)	0.98 (0.88–1.11)	0.9 (0.81–1.01)	0.86 * (0.77–0.95)	0.87 * (0.79–0.97)	1.05 (0.96–1.15)	0.93 (0.83–1.04)	0.93 (0.74–1.16)
High Blood Cholesterol	1.13 * (1.03–1.25)	1.16 * (1.03–1.3)	1.07 (0.96–1.19)	0.83 * (0.75–0.91)	0.93 (0.84–1.02)	0.89 * (0.8–0.98)	0.84 ** (0.78–0.91)	1.19 * (1.08–1.32)	0.86 (0.7–1.06)

* Statistical significance at *a* = 0.05 significance level, ** statistical significance at *a*’ = 0.00017 level after Bonferroni correction.

## Supplementary Materials

The following are available online at https://www.mdpi.com/2072-6643/12/7/2148/s1, Figure S1: Graphical representation of the frequencies of responses from the participants by food group and FFQ quartiles of baseline consumption during the first FU-DQ period. Figure S2: Graphical representation of the frequencies of responses from the participants by food group and FFQ quartiles of baseline consumption during the second FU-DQ period. Table S1: Number and percentages (%) of participants’ responds regarding consumption of vegetables, fruit, legumes, cereals, dairy products, red meat and fish/seafood, olive oil and nuts compared to their corresponding intake at recruitment, by FU-DQ period. The EPIC-Greece study.
