# Supplementary material for: Changes in the Dietary Habits of the Greek EPIC Cohort Participants during a 14-Year Follow-Up Period (1997–2011)
Source: Nutrients. 2020 Jul 19;12(7):2148. doi: 10.3390/nu12072148 (PMC7400902; doi:10.3390/nu12072148)
Supplement: Supplementary file 1 [file nutrients-12-02148-s001.pdf]

nutrients

MDPI

Original Article- Supplementary materials

Supplementary materials

Changes in the dietary habits of the Greek EPIC cohort participants during a 14-year follow-up period (1997-2011)

Nikolaos Skourlis*[1], Ioannis Patsis[1], Georgia Martimianaki[1], Eleni Peppa[1], Antonia Trichopoulou[1], Klea Katsouyanni[2]

[1] Hellenic Health Foundation; info@hhf-greece.gr
[2] Department of Epidemiology and Biostatistics, University of Athens

Figures S1 and S2 are stacked bar plots presenting the proportion of each answer regarding the consumption of participants compared to the baseline of the study, by FFQ (food frequency questionnaire) quartiles of baseline consumption over food group (Vegetables, Legumes, Fruits, Dairy products, Nuts, Cereals, Meat and meat products, Fish/Seafood and Olive oil) for the 1st FU-DQ (Figure S1) and the 2nd FU-DQ (Figure S2). The answers are grouped as ""More"/Much "More"", "Same", "Less/Much less" and "None/Do not remember".

Table S1 shows in detail the frequency of each FU-DQ answer category ("Much more", "More", "Same", "Less", "Much Less", "None", "Do not Remember") regarding consumption of vegetables, fruit, legumes, cereals, dairy products, meat and fish/seafood, olive oil and nuts comparative to their corresponding intake at recruitment, by FU-DQ period.

Figure S1. Graphical representation (are stacked bar plot) of the frequencies of responses from the participants by food group and FFQ quartiles of baseline consumption during the 1st FU-DQ period

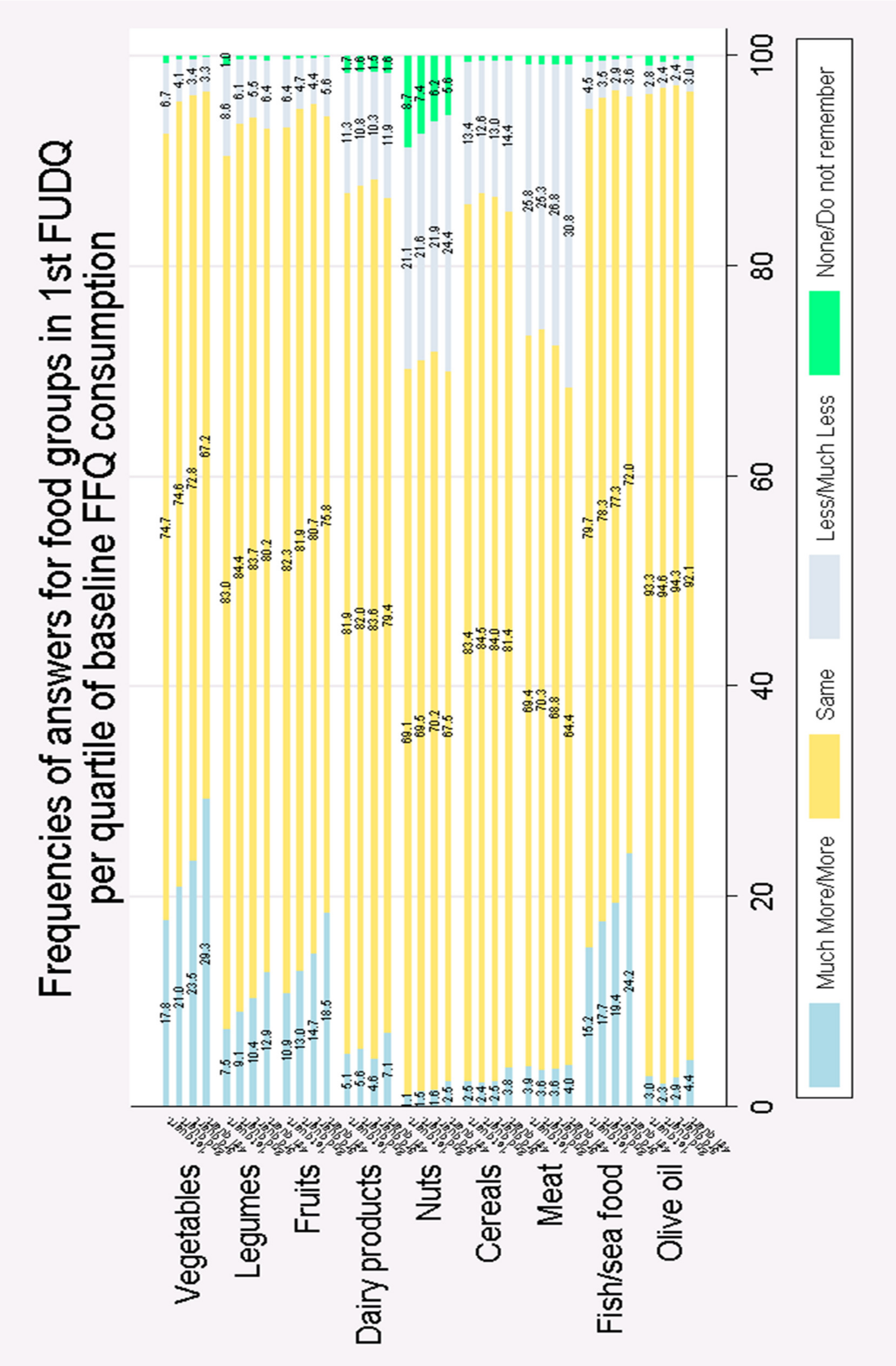

Proportions less than 1% are not depicted with numbers

Figure S2. Graphical representation of the frequencies of responses from the participants by food group and FFQ quartiles of baseline consumption during the 2nd FU-DQ period

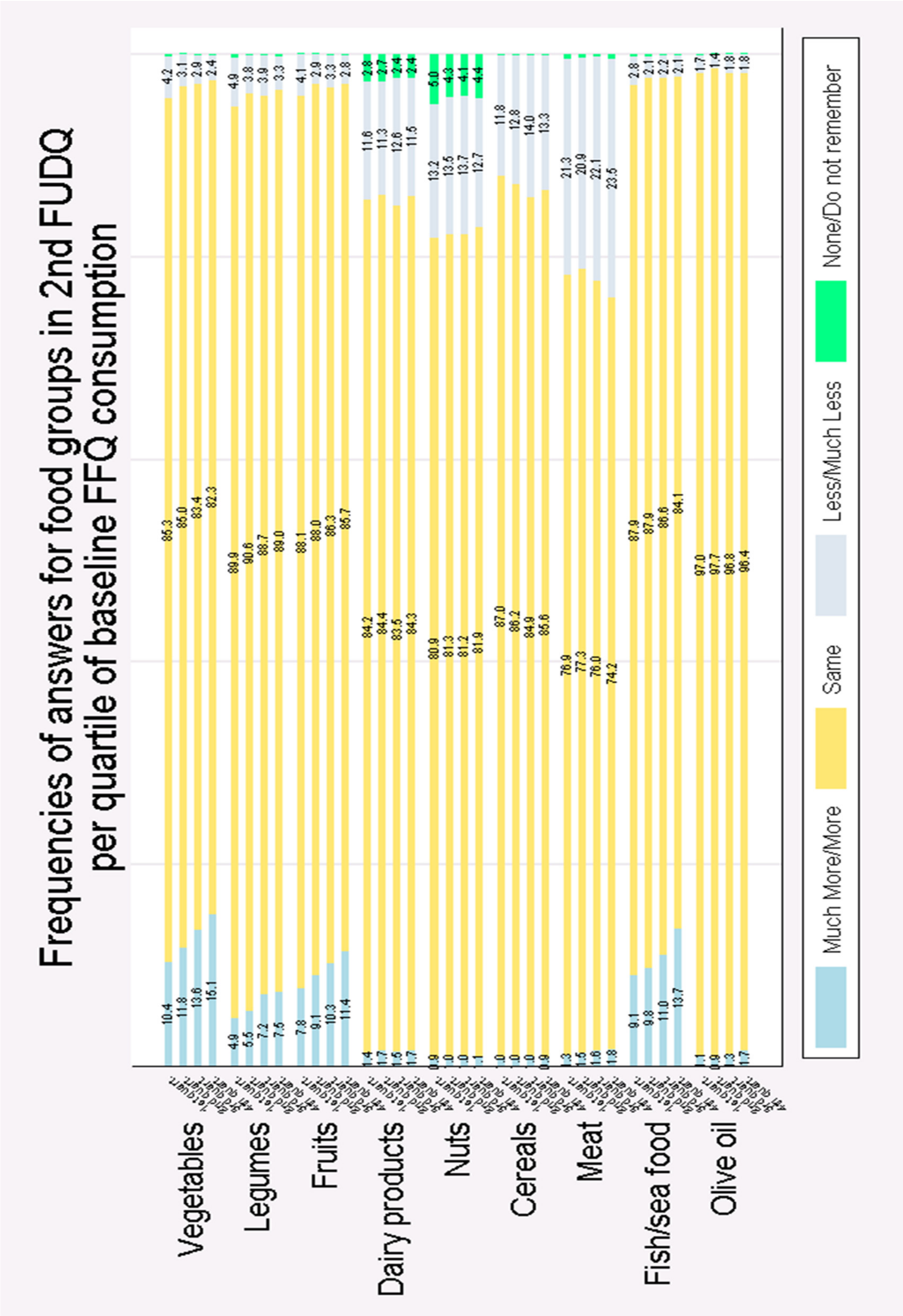

Proportions less than 1% are not depicted with numbers

Table S1. Number and percentages (%) of participants' responds regarding consumption of vegetables, fruit, legumes, cereals, dairy products, red meat and fish/seafood, olive oil and nuts comparative to their corresponding intake at recruitment, by FU-DQ period. The EPIC-Greece study.

Food item/group	Period FUDQ	Much More	More	Same	Less	Much less	None	Do not Remember	Total
		N(%)	N(%)	N(%)	N(%)	N (%)	N(%)	N(%)	
Vegetables	1	278 (1.25)	4812 (21.67)	16070 (72.35)	764 (3.44)	201 (0.9)	68 (0.31)	17 (0.08)	22210
	2	117 (0.63)	2243 (12.16)	15487 (83.94)	429 (2.33)	148 (0.8)	24 (0.13)	2 0.01)	18450
Cereals	1	13 (0.06)	604 (2.72)	18529 (83.43)	2333 (10.5)	616 (2.77)	92 (0.41)	23 (0.1)	22210
	2	6 (0.03)	176 (0.95)	15846 (85.89)	1972 (10.69)	425 (2.3)	23 (0.12)	2 (0.01)	18450
Legumes	1	82 (0.37)	2140 (9.64)	18404 (82.86)	1130 (5.09)	328 (1.48)	106 (0.48)	20 (0.09)	22210
	2	29 (0.16)	1129 (6.12)	16519 (89.53)	563 (3.05)	171 (0.93)	37 (0.2)	2 (0.01)	18450
Fruits	1	195 (0.88)	2981 (13.42)	17803 (80.16)	956 (4.3)	212 (0.95)	46 (0.21)	17 (0.08)	22210
	2	73 (0.4)	1711 (9.27)	16052 (87)	504 (2.73)	96 (0.52)	12 (0.07)	2 (0.01)	18450
Dairy products	1	50 (0.23)	1185 (5.34)	18147 (81.71)	1963 (8.84)	490 (2.21)	356 (1.6)	19 (0.09)	22210
	2	9 (0.05)	280 (1.52)	15521 (84.12)	1650 (8.94)	519 (2.81)	469 (2.54)	2 (0.01)	18450
Meat	1	20 (0.09)	820 (3.69)	15164 (68.28)	4881 (21.98)	1148 (5.17)	160 (0.72)	17 (0.08)	22210
	2	3 (0.02)	282 (1.53)	14033 (76.06)	3390 (18.37)	665 (3.6)	75 (0.41)	2 (0.01)	18450
Fish/ Seafood	1	253 (1.14)	3976 (17.9)	17085 (76.92)	649 (2.92)	155 (0.7)	75 (0.34)	17 (0.08)	22210
	2	65 (0.35)	1954 (10.59)	15971 (86.56)	345 (1.87)	81 (0.44)	32 (0.17)	2 (0.01)	18450
Nuts	1	13 (0.06)	360 (1.62)	15351 69.12)	3309 (14.9)	1634 (7.36)	1514 (6.82)	29 (0.13)	22210
	2	5 (0.03)	175 (0.95)	14999 (81.3)	1631 (8.84)	827 (4.48)	811 (4.4)	2 (0.01)	18450
Olive oil	1	116 (0.52)	606 (2.73)	20769 (93.51)	467 (2.1)	117 (0.53)	121 (0.54)	14 (0.06)	22210
	2	5 (0.03)	227 (1.23)	17893 (96.98)	263 (1.43)	50 (0.27)	10 (0.05)	2 (0.01)	18450
